# Association between annual concentration of air pollutants and incidence of metabolic syndrome among Korean adults: Korean Genome and Epidemiology Study (KoGES)

**DOI:** 10.1186/s12940-025-01158-7

**Published:** 2025-02-11

**Authors:** Hanuel Shin, Minkyo Song, Sanghyuk Bae

**Affiliations:** 1 Graduate School of Public Health and Healthcare Management, The Catholic University of Korea, Seoul, Republic of Korea; 2 Department of Nursing, Seoul St. Mary’s Hospital, College of Medicine, The Catholic University of Korea, Seoul, Republic of Korea; 3 Immunoepidemiology Unit, Laboratory of Epidemiology and Population Sciences, National Institute on Aging, National Institutes of Health, Bethesda, USA; 4 Department of Preventive Medicine, College of Medicine, The Catholic University of Korea, 222, Banpo-daero, Seocho-gu, Seoul, 06591 Republic of Korea

**Keywords:** Air pollutants, Adult, Cohort studies, Incidence, Metabolic syndrome

## Abstract

**Background:**

Air pollution is a global public health concern and incidence rates of metabolic syndrome (MetS) are increasing. To evaluate the effect of long-term air pollution exposure, we examined the association between long-term exposure to ambient air pollution and the incidences of MetS among Korean adults.

**Methods:**

We used data from the Korean Genome and Epidemiology Study’s Cardiovascular Disease Association Study, a population-based cohort consisting of community-dwelling Korean adults between 2005 and 2011, who were followed up with until 2016 (*n* = 7,428). Air pollution exposure was estimated using the Congestion Mitigation and Air Quality model based on the participants’ addresses. The participants had a physical examination at every visit during follow-up, and MetS was defined based on the National Institute of Health’s National Cholesterol Education Program-Adult Treatment Panel III. We used Cox proportional hazard model to analyze the association between long-term air pollution exposure and incidences of MetS per interquartile range (IQR) increment of the annual concentration after adjusting for potential confounders using single and two-pollutant analysis.

**Results:**

The hazard ratios (HR) of MetS per IQR increment in PM_2.5_, SO_2_, NO_2_, and CO were 1.19 (95% CI: 1.12-1.27), 1.57 (95% CI: 1.47-1.68), 1.11 (95% CI: 1.03-1.20), and 1.63 (95% CI: 1.48-1.78), respectively. The incidences of MetS components, which are high blood pressure, elevated fasting glucose, abdominal obesity, high fasting triglyceride (TG), and low fasting high-density lipoprotein (HDL-C), were significantly associated with an IQR increment especially in SO_2_ and CO. In subgroup analysis, males had higher risk of MetS than females. The HR was the highest in the 60–69 year old age group for all pollutants.

**Conclusion:**

In the present study, we found that long-term ambient air pollution exposure increased the incidences of MetS and its components among Korean adults, especially in males and the elderly population.

**Graphical abstract:**

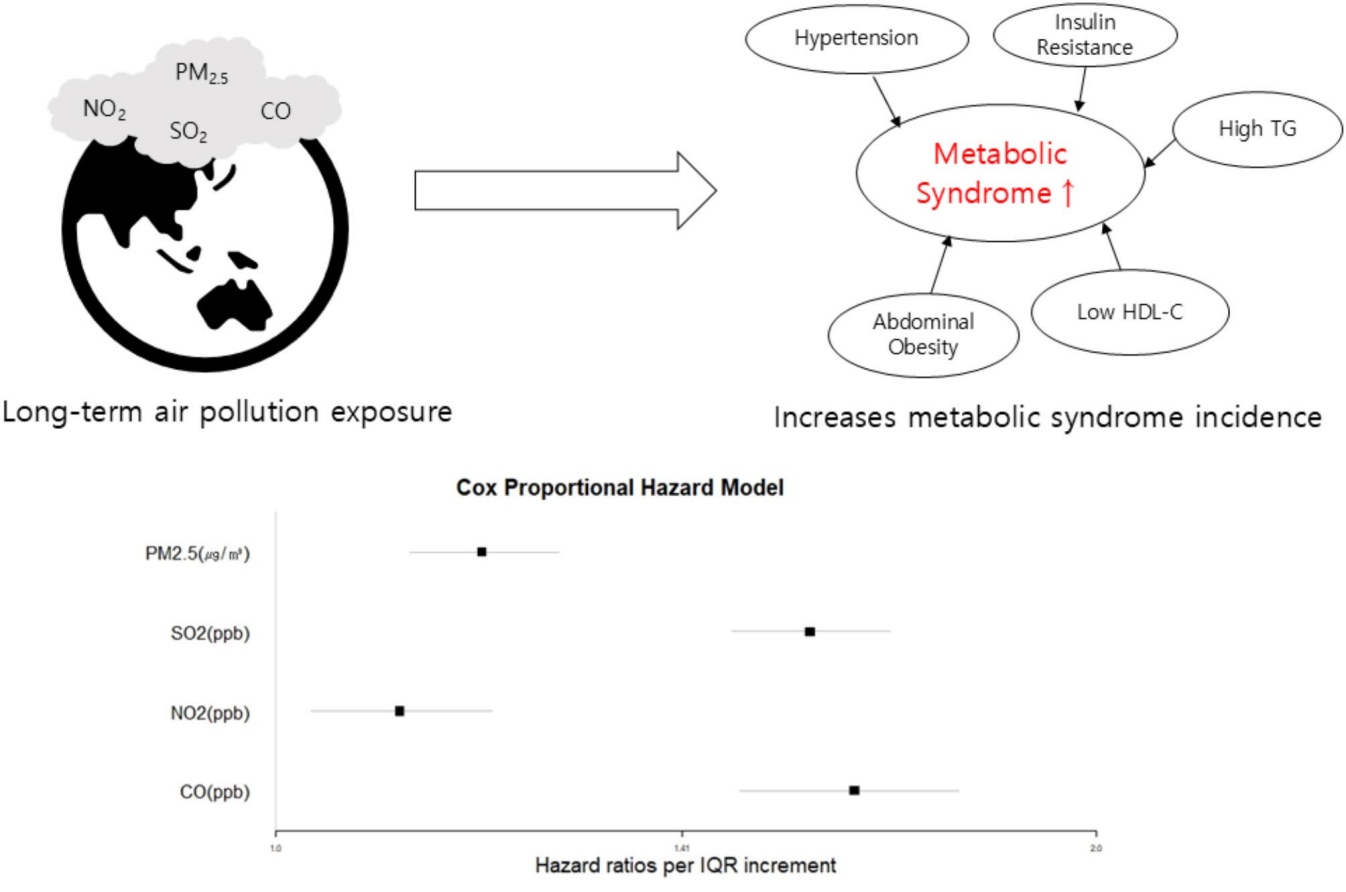

**Supplementary Information:**

The online version contains supplementary material available at 10.1186/s12940-025-01158-7.

## Background

Metabolic syndrome (MetS) is defined as the presence of three or more of the following symptoms: high blood pressure, elevated fasting glucose, abdominal obesity, high fasting triglyceride (TG) level, and low fasting high-density lipoprotein cholesterol (HDL-C) levels. MetS affects quality of life, leading to chronic diseases, such as cardiovascular disease, cancer, and diabetes [1–3]. The prevalence of MetS globally varied from 12.5% (95% CI: 10.2-15.0) to 31.4% (95% CI: 29.8-33.0) depending on ethnic or cultural background [4]. In year 2011–2012, the prevalence of MetS among American adults was 32.5% and increased to 36.9% in year 2015–2016 [5].

Air pollution is ambient atmospheric contamination due to chemical substances, gases, or particulate matter. The pollutants majorly affecting human health are particulate matter (PM) and gaseous substances, such as carbon monoxide (CO), ozone (O_3_), nitrogen dioxide (NO_2_), and sulfur dioxide (SO_2_), and these pollutants form through human activity and natural sources [6]. Most air pollutants originate from human activities, such as transportation, industrial and agricultural activities, power plants, residential heating and cooking, and combustion of fossil fuels [7, 8]. Air pollution exposure affects almost all the world’s population. The World Health Organization (WHO) estimated that 99% of the world’s population was exposed to air pollutants that exceeded the WHO standards in 2019 [6]. As of 2019, air pollution is the fourth leading cause of death worldwide, and 667 million people die annually from air pollution [9, 10]. Although the concentration of air pollutants in Korea is generally decreasing due to environmental regulations, it is still higher than the environmental standards of Korea or the WHO [6].

Although the main risk factor for MetS is unhealthy lifestyle patterns [11, 12], previous studies have shown that ambient air pollution exposure is also a risk factor for MetS [13, 14]. Thus far, studies have shown a strong association between PM exposure and MetS incidences, especially from long-term exposure in both younger and older populations [15–19]. A meta-analysis study showed that 5 µg/m^3^ increase in the annual PM_2.5_ or PM_10_ concentration increases MetS risks by 14% and 9%, respectively [20]. Gaseous substances, such as SO_2_, NO_2_, and O_3_, also showed a significant increase in MetS risks from long-term exposures [21–23]. The prevalence of MetS components, such as hypertension and hyperglycemia, also showed a significant association with air pollution in previous studies conducted in Korea [24, 25] and other countries [26–29].

However, the evidence of the association between long-term air pollution exposure and MetS is still limited in terms of study design and pollutant models. For study design, most of the previous studies were conducted using cross-sectional datasets, and more longitudinal studies are needed. There have not yet been many studies conducted using longitudinal dataset in Korea, assessing air pollutant exposure and MetS incidence. Due to the regional differences in the composition of air pollutants in geographical context, more longitudinal studies are needed in Korea. In terms of pollutant models, previous studies analyzed the association between air pollution and MetS incidence using a single pollutant model, mostly using PM. To the best of our knowledge, there has not yet been a study that analyzed the association between PM, gaseous substances and Mets incidence for a longer duration, using a population-based dataset. Two- or multi-pollutant analysis is used to estimate the independent effect between the outcome disease and each pollutant after adjusting for potential confounders [30]. Thus, it is necessary to assess the association between the long-term effects of air pollution and MetS incidences using two- or multi-pollutant models, in order to isolate the effect of each pollutant and to refine the environmental regulations.

Therefore, we aimed to analyze the association between long-term air pollution exposure and the incidences of MetS among Korean adults using the dataset from the Korean Genome and Epidemiology Study (KoGES) cohort, a large-scale population-based cohort study conducted in the long term, using both single and two-pollutant model, in the present study.

## Methods

### Study population

We used the dataset of the KoGES cohort with estimated air pollution data. The KoGES is a population-based prospective cohort study conducted by the National Institute of Health, Korea Disease Control and Prevention Agency [31]. Cohort construction and detailed descriptions were provided in a previous study [32]. Among the KoGES sub-studies, we used the KoGES Cardiovascular disease Association Study (CAVAS), which consists of community-dwelling Korean adults between the ages of 40 and 69, in the present study. The purpose of this sub-study was to identify the risk factors related to the development of cardiovascular diseases and establish measures for disease prevention and early diagnosis, by investigating the impact of lifestyle, diet, and environmental factors on the development of chronic diseases in community-dwelling populations. In addition, CAVAS covers the largest regions, which may be more suitable for generalizing the study results. The six study areas were Yangpyeong (area 877.1 km^2^), Namwon (area 752.6 km^2^), Goryeong (area 384.0 km^2^), Wonju (area 867.3 km²), Pyeongchang (area 1,464.1 km²), and Ganghwa (area 411.4km^2^). Figure S1 shows the geographical location of six study regions. In baseline and every follow-up visits, same protocol was used by providing identical questionnaires, physical examinations, and clinical investigations. Trained interviewers interviewed the participants regarding the sociodemographic status, lifestyle patterns such as smoking, drinking and physical activity, diet, disease, family history and health conditions. Participants’ blood and urine samples were collected by trained technicians according to the standard procedures [32].

The baseline survey was conducted between 2005 and 2011, and follow-ups were conducted from 2007 to 2016 in six regions. The first follow-up survey was completed within 36 months after the baseline survey, the second within 4–6 years after the baseline survey, the third within 7–9 years after the baseline survey, and the fourth within 10–12 years after the baseline survey. A detailed timeline of recruitment and follow-up survey was presented in Figure S2. An event was defined as the concurrent occurrence of three or more components of MetS after cohort enrollment.

**Fig. 1:**
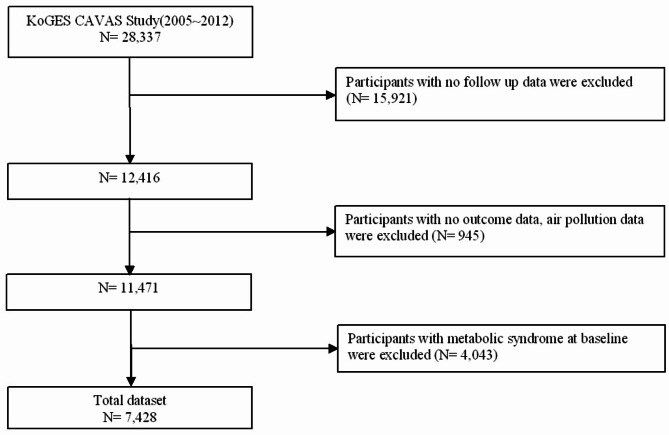
Flowchart of study population selection

The KoGES CAVAS enrolled 28,337 participants at baseline. Among the 28,337 participants, those without follow-up data, those without physical assessment data to diagnose MetS, those without air pollution exposure data, and those with MetS at the baseline were excluded. In total, 7,428 participants were included in the current analysis (Fig. 1). The present study was approved by the Institutional Review Board at College of Medicine, The Catholic University of Korea (No.MC22ZASI0062).

### Exposure assessment

The air pollutants measured were PM ≤ 2.5 microns in diameter (PM_2.5_), SO_2_, NO_2_, CO, and O_3_. Air pollution concentrations were calculated using the United States Environmental Protection Agency (US EPA)’s Model-3 Congestion Mitigation and Air Quality (CMAQ) version 4.7.1. The CMAQ model included a meteorological, emission model, and chemical transport model. Using the CMAQ model combined with satellite-derived aerosol optical depth (AOD), the concentrations of PM were estimated in a 1 km by 1 km grid. The concentrations of the gaseous pollutants were estimated in a 9 km by 9 km grid. Multiple linear regression (MLR) was additionally applied for PM and O_3_ to further validate the results. According to a meta-analysis study that analyzed the source apportionment (SA) of PM by regions, the SA of PM in Yangpyeong and Ganghwa was highest for secondary aerosol (including secondary nitrate and secondary sulfate) and motor vehicle, while Namwon was highest for motor vehicle and secondary aerosol. In Wonju and Pyeongchang, SA was not accurately known as no studies were conducted [33]. The verification of the modeling data was conducted by comparing it with the monitoring station data. The square of the correlation coefficient (R^2^) values on a daily scale were 0.66 for PM_2.5_, 0.74 for NO_2_, and 0.69 for CO. The description of the air pollution modeling methods was provided previously [34]. The participants’ addresses were geocoded using the geocoding software GeoService-Xr (Geoservice, Seoul, Republic of Korea) into latitude and longitude, and matched to the center of the estimated air pollutant concentration grid. If a participant’s address was changed due to relocation between follow-up surveys, the average exposure was calculated assuming that the participant moved at the midpoint between the previous and current follow-up survey.

In this present study, the association between annual air pollution exposure and MetS incidences was analyzed using the average annual concentration data of PM_2.5_, NO_2_, CO, SO_2_, and O_3_. To observe the personal cumulative long-term air pollution exposure concentration, personal annual average exposure was calculated from 1 year prior to the study enrollment to the occurrence of MetS or to the point when the participant is censored. Personal exposure assessment calculation was presented in Figure S2.

### Definition of metabolic syndrome (MetS)

MetS was diagnosed using the participants’ physical examination data, specifically height, weight, and blood and urine samples. We defined MetS cases according to the National Institute of Health (NIH)’s National Cholesterol Education Program-Adult Treatment Panel III (NCEP-ATP III). Individuals with three or more of the symptoms noted below were diagnosed as MetS [35]. Components of MetS include high blood pressure (systolic ≥ 130 mmHg, diastolic ≥ 85 mmHg, or taking antihypertension medication), elevated fasting glucose (fasting blood glucose ≥ 100 mg/dL), abdominal obesity (waist circumference ≥ 90 cm in male, ≥ 80 cm in female), fasting TG (≥ 150 mg/dL), or fasting HDL-C (< 40 mg/dL in males, < 50 mg/dL in females).

### Covariates

We adjusted the sociodemographic variables, lifestyle characteristics, and meteorological variables. Sociodemographic characteristics included age, sex, region, occupational status, monthly household income level, education level, and family history of hypertension and hyperglycemia. The education level was grouped into four categories: ‘Elementary school,’ ‘Middle school’, ‘High school’, and ‘Over college’. The monthly household income level was classified into four groups: ‘<1,000,000 won/month’, ‘1,000,000–1,990,000 won/month’, ‘2,000,000–3,990,000 won/month’, and ‘≥4,000,000 won/month’ in Korean Won (KRW) monthly. Occupational status, and family history of hypertension and hyperglycemia were grouped into three categories, which were ‘yes’, ‘no’, and ‘unknown’. If the covariate variable was not surveyed (the data was missing, specific year or specific unit), then the answer was classified as ‘unknown’.

Lifestyle characteristics were alcohol consumption, smoking status, and physical activity. Alcohol consumption and smoking status were categorized into ‘current’, ‘past’, and ‘never’. As for physical activity, it was classified into ‘1–2 times a week’, ‘3–6 times a week’, ‘every day’, and ‘no’.

BMI was calculated using height and weight from physical examination data by dividing weight (kg) by the square of height (m). We used the criteria for the Asian population by the WHO for which the values were classified into five groups [36]. The categories were underweight (BMI < 18.5 kg/m^2^), normal (18.5–22.9 kg/m^2^), overweight (23.0–24.9 kg/m^2^), obese I (25.0–29.9 kg/m^2^), and obese II (BMI $\geq$ 30 kg/m^2^).

We adjusted the average annual temperature (℃) and relative humidity (%) to adjust the regional and climate effects. Based on previous studies, we designed a directed acyclic graph (DAG) using the software DaGitty [37]. DAG was presented in Figure S3.

### Statistical analysis

We analyzed the distribution of the sociodemographic characteristics using descriptive statistics. The Spearman correlation coefficients were estimated between the air pollutants and meteorological variables. The mean (± standard deviation), median, minimum, maximum, and interquartile range (IQR) of the air pollutants exposure concentrations during the follow-up were presented. We used Cox proportional hazard model to analyze the association between long-term air pollution exposure per IQR increment and the incidences of MetS, and the results were presented as hazard ratios (HRs) and 95% confidence intervals (CIs). Also, to check for the independent effect of each pollutant, we performed two-pollutant model between PM_2.5_ and NO_2_, SO_2_, and O_3_ respectively. We used age as the timescale when performing Cox regression by using the age at which MetS occurred or when censoring happened. This method is used when the covariate of interest is strongly associated with age, and since age is a major risk factor for MetS, we assumed this method was suitable. Censoring was defined as those who did not have MetS until the end of the study period, or a follow-up loss due to migration or drop out. An individual’s follow-up period was defined as from the time of the baseline study to the point when MetS was diagnosed, or the time between the baseline survey and censoring. Model 1 was an unadjusted model, and Model 2 was adjusted for age and sex. In addition to model 2 covariates, Model 3 was adjusted for sociodemographic and lifestyle characteristics, which were monthly household income, education level, smoking and drinking status, physical activity. Model 4, which was our main model, was additionally adjusted for temperature and humidity. Stratified analyses by sex (male, female), age groups (40–49 years, 50–59 years, 60–69 years, 70 years older), and smoking status (current, past, never, unknown) were performed to find the association between subgroups. Sensitivity analysis was conducted by adding region, and BMI to the main model, respectively. This sensitivity analysis allowed us to observe whether region act as a confounder and BMI as an intermediate. MetS is diagnosed when concurrent occurrence of the component diseases, and the association between air pollution and MetS component diseases may vary. Therefore, we also analyzed the association between MetS components, which were hypertension, hyperglycemia, abdominal obesity, high TG, and low HDL-C, and air pollution exposure. When performing analyses for each component, we excluded participants with abnormal symptoms at baseline. We used R (version 4.2.3) and a two-tailed *P*-value less than 0.05 to determine the statistical significance.

## Results

### Descriptive statistics

The cohort was comprised of 7,428 participants and a total of 37,780 person-years of follow-up. The mean follow-up duration was 5 years (1-10.8 years) and there were 1,773 new MetS cases during the follow-up period. The average age at the time of enrollment was 58.2 years old (± 9.6), and 51.2% of the participants were male. A majority of the study participants had a job (87.9%), an education level of elementary school (48.3%), never consumed alcohol (46.5%), never smoked (52.0%), and did not exercise regularly (65.8%) (Table 1). We compared the baseline characteristics of those who excluded and included. The excluded were older (58.7 years) and 66.4% was female. The excluded group included those with MetS at baseline, so the physical examination and biochemistry results were either higher or worse than that of study population. The average air pollution exposure value was also compared. The participants who were included in the study had higher exposure value for all six air pollutants. In some cases, air pollution values were missing for the excluded, which may explain the greater exposure values for those included in the study (Table S1). The population with new cases of MetS were older (58.6 years), had more female (57.2%) and evenly distributed in study regions (Table S2). Table S3 shows the distribution of MetS and non-MetS cases by each air pollutant variable by quartiles. In this analysis, the MetS incidence among participants living in regions with higher air pollution was higher. Year 2008 had the greatest number of MetS incidences in all regions, followed by year 2011 in Yangpyeong, Pyeongchang, and Ganghwa (Table S4).

**Table 1:** Baseline descriptive characteristics of study population (2005–2012)

Characteristics	Value (*n* = 7,428)
Age, year, mean ± SD	58.2 ± 9.6
Sex, n (%)	
Male	3804 (51.2)
Female	3624 (48.8)
Residence, n (%)	
Yangpyeong	1411 (17.7)
Namwon	1109 (14.9)
Goryeong	1215 (16.4)
Wonju	1278 (17.2)
Pyeongchang	1029 (13.9)
Ganghwa	1486 (20.0)
Monthly household income, KRW, n (%)	
< 1,000,000 won/month	1260 (17.0)
1,000,000 ~ 1,990,000 won/month	1005 (13.5)
2,000,000 ~ 3,990,000 won/month	1030 (13.9)
≥ 4,000,000 won/month	304 (4.1)
Unknown	3829 (51.6)
Job status, n (%)	
Yes	6528 (87.9)
No	287 (3.9)
Unknown	613 (8.3)
Education, n (%)	
Elementary school	3585 (48.3)
Middle school	1382 (18.6)
High school	1697 (22.9)
Over college	747 (10.1)
Unknown	17 (0.2)
Drinking status, n (%)	
Current	3482 (46.9)
Past	485 (6.5)
Never	3454 (46.5)
Unknown	7 (0.1)
Smoking status, n (%)	
Current	1112 (15.0)
Past	1286 (17.3)
Never	3863 (52.0)
Unknown	1167 (15.7)
Physical activity, n (%)	
1–2 times a week	565 (7.6)
3–6 times a week	1080 (14.5)
Everyday	868 (11.7)
No	4890 (65.8)
Unknown	25 (0.3)
BMI, n (%)	
< 18.5 kg/m²	184 (2.5)
18.5–22.9 kg/m²	2915 (39.2)
23.0–24.9 kg/m²	1991 (26.8)
25.0–29.9 kg/m²	2338 (31.5)
≥ 30 kg/m²	0
Family history of Hypertension, n (%)	
Yes	1518 (20.4)
No	5855 (78.8)
Unknown	55 (0.7)
Family history of Hyperglycemia, n (%)	
Yes	934 (12.6)
No	6443 (86.8)
Unknown	51 (0.7)
Waist circumference, cm, mean ± SD	82.7 ± 8.8
Systolic blood pressure, mmHg, mean ± SD	121.5 ± 16.5
Diastolic blood pressure, mmHg, mean ± SD	77.3 ± 10.5
Fasting blood glucose, mg/dL, mean ± SD	94.7 ± 18.9
Total cholesterol, mg/dL, mean ± SD	195.4 ± 34.9
Triglyceride, mg/dL, mean ± SD	123.1 ± 74.3
High density lipoprotein cholesterol, mg/dL, mean ± SD	46.6 ± 11.4

Note: Data were shown as mean ± SD for continuous variables and number (%) for categorical variables

Abbreviations: SD, Standard deviation; KRW, Korean won; BMI, Body mass index

The annual averages of air pollutants and meteorological variables during follow-up are shown in Table 2. The annual mean concentrations of PM_2.5_ was 26.86 µg/m^3^, which was higher than the WHO standards [38]. The IQR values of air pollutant exposure during follow-up were 3.48 µg/m^3^ for PM_2.5_, SO_2_ for 1.17 ppb, NO_2_ for 8.87 ppb, CO for 172.71 ppb, and O_3_ for 2.65 ppb. The average temperature during follow-up was 10.65℃, and the average relative humidity was 71.97%. The correlation coefficients were highest between SO_2_ and CO which was 0.88. There was a negative correlation between O_3_ and other air pollutants, and temperature was negatively correlated with air pollutants. Relative humidity was positively correlated with NO_2_ and O_3_, and negatively correlated with other air pollutants (Figure S4).

**Table 2:** Average annual concentration of air pollutants and meteorological exposure during follow-up

Exposure	Mean ± SD	Median	Minimum	Maximum	IQR
PM_2.5_ (µ/m^3^)	26.86 ± 2.74	26.59	17.91	35.67	3.48
SO_2_ (ppb)	4.37 ± 0.94	4.30	2.34	8.75	1.17
NO_2_ (ppb)	15.30 ± 5.31	15.05	4.98	36.77	8.87
CO (ppb)	487.86 ± 98.22	468.00	288.35	725.73	172.71
O_3_ (ppb)	25.39 ± 2.167	25.09	18.04	37.36	2.65
Temperature (℃)	10.65 ± 1.48	10.75	6.50	13.64	2.28
Humidity (%)	71.97 ± 1.34	71.99	61.70	76.72	1.62

Abbreviations: SD, Standard deviation; IQR, Interquartile range; PM_2.5_, particulate matter with aerodynamic diameters ≤ 2.5 μm; SO_2_, sulfur dioxide; NO_2_, nitrogen dioxide; CO, carbon monoxide; O_3_, ozone; ppb, parts per billion

### Long-term exposure to ambient air pollution and incidence of metabolic syndrome and its component

Table 3 shows the HRs (95% CIs) for the incidences of MetS per IQR increment of air pollution exposure. Exposure to ambient air pollution was significantly associated with a higher risk of incidences of MetS except for O_3_. From Model 4, which was our main model, the HR of MetS per IQR increment for PM_2.5_, SO_2_, NO_2_, and CO were 1.19 (95% CI: 1.12-1.27), 1.57 (95% CI: 1.47-1.68), 1.11 (95% CI: 1.03-1.20), and 1.63 (95% CI: 1.48-1.78), respectively.

**Table 3:** Hazard ratios of metabolic syndrome per interquartile range width increment in air pollution exposure during follow-up (*n* = 7,428)

Exposure	Model 1^a^	Model 2^b^	Model 3^c^	Model 4^d^
PM_2.5_ (µg/m^3^)	1.19(1.11–1.26)	1.17 (1.10–1.24)	1.17 (1.10–1.25)	1.19 (1.12–1.27)
SO_2_ (ppb)	1.48(1.40–1.58)	1.55 (1.45–1.64)	1.71 (1.60–1.83)	1.57 (1.47–1.68)
NO_2_ (ppb)	1.16(0.73–1.83)	1.22 (1.13–1.32)	1.20 (1.11–1.30)	1.11 (1.03–1.20)
CO (ppb)	1.53(1.41–1.67)	1.59 (1.46–1.73)	1.68 (1.54–1.84)	1.63 (1.48–1.78)
O_3_ (ppb)	0.71(0.67–0.76)	0.47 (0.44–0.51)	0.42 (0.39–0.45)	0.48 (0.45–0.52)

Note: IQR for PM_2.5_: 3.48 µg/m^3^, SO_2_: 1.17 ppb, NO_2_: 8.87 ppb, CO: 172.71 ppb, O_3_: 2.65 ppb

Abbreviations: HR, Hazard ratio; 95% CI, 95% confidence interval; PM_2.5_, particulate matter with aerodynamic diameters ≤ 2.5 μm; SO_2_, sulfur dioxide; NO_2_, nitrogen dioxide; CO, carbon monoxide; O_3_, ozone; ppb, parts per billion

^a^Model 1 Unadjusted model

^b^Model 2 adjusted for age, sex

^c^Model 3 adjusted for age, sex, monthly household income, education, smoking, drinking, physical activity

^d^Model 4 adjusted for age, sex, monthly household income, education, smoking, drinking, physical activity, temperature, humidity

Results of two-pollutant model are presented in Table S5. We performed two-pollutant analysis for PM_2.5_ with SO_2_, NO_2_, and O_3_. After adjusting for PM_2.5_, effect for NO_2_ was not statistically significant (HR: 0.90, 95% CI: 0.80-1.01). However, the HR of PM_2.5_ was 1.27 (95% CI: 1.16-1.39), remaining significant after adjusting for NO_2_. In two-pollutant analysis, SO_2_ was more strongly associated with the outcome in comparison to each individual compound. When combined with PM_2.5_, SO_2_ remained significant, showing the HR of 1.75 (95% CI: 1.60-1.91) per IQR increment.

Similar results were found for incidences of MetS components. The HRs (95% CIs) for the incidences of MetS components per IQR increment of air pollution exposure are shown in Table 4. When adjusted for potential confounders, the risk increased the highest for SO_2_ and CO.

**Table 4:** Hazard ratios of metabolic syndrome components per interquartile range width increment in air pollution exposure during follow-up

Exposure	Hypertension	Hyperglycemia	Abdominal obesity	High TG	Low HDL-C
	(*n* = 5,208)	(*n* = 7,745)	(*n* = 6,948)	(*n* = 7,358)	(*n* = 6,616)
PM_2.5_ (µ/m^3^)	1.19 (1.11–1.27)	1.16 (1.09–1.23)	1.30 (1.23–1.39)	1.10 (1.04–1.17)	1.47 (1.34–1.61)
SO_2_ (ppb)	1.44 (1.34–1.55)	1.32 (1.24–1.41)	1.67 (1.57–1.79)	1.35 (1.27–1.43)	1.86 (1.67–2.07)
NO_2_ (ppb)	1.05 (0.96–1.14)	0.96 (0.89–1.04)	1.40 (1.30–1.52)	1.07 (1.00-1.15)	1.46 (1.29–1.65)
CO (ppb)	1.44 (1.30–1.59)	1.44 (1.32–1.57)	1.79 (1.64–1.96)	1.38 (1.27–1.50)	1.85 (1.61–2.13)
O_3_ (ppb)	0.51 (0.48–0.56)	0.48 (0.44–0.51)	0.50 (0.46–0.54)	0.54 (0.51–0.58)	0.44 (0.39–0.49)

Note: IQR for PM_2.5_: 3.48µ/m^3^, SO_2_: 1.17ppb, NO_2_: 8.87ppb, CO: 172.71ppb, O_3_: 2.65ppb

Abbreviations: HR, Hazard ratio; 95% CI, 95% confidence interval; PM_2.5_, particulate matter with aerodynamic diameters ≤ 2.5 μm; SO_2_, sulfur dioxide; NO_2_, nitrogen dioxide; CO, carbon monoxide; O_3_, ozone; ppb, parts per billion

All models adjusted for age, sex, monthly household income, education, smoking, drinking, physical activity, temperature, humidity

### Subgroup analysis

**Fig. 2:**
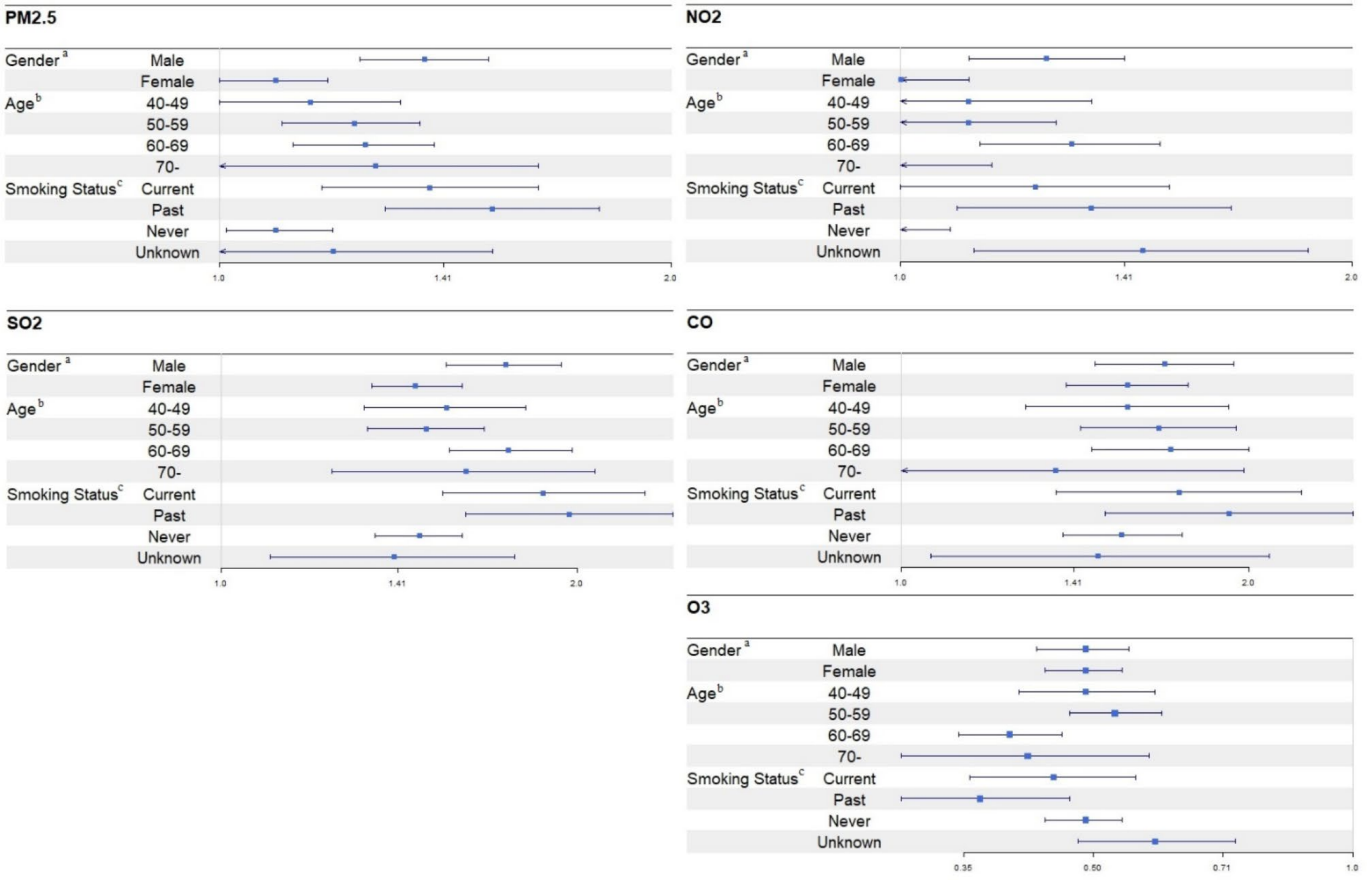
Hazard ratios of metabolic syndrome per interquartile range width increment in air pollution exposure during follow-up stratified by sex, age, and smoking status (*n*=7,428). Note: IQR for PM_2.5_: 3.48 µg/m^3^, SO_2_: 1.17 ppb, NO_2_: 8.87 ppb, CO: 172.71 ppb, O_3_: 2.65 ppb. Abbreviations: PM_2.5_, particulate matter with aerodynamic diameters ≤ 2.5 μm; SO_2_, sulfur dioxide; NO_2_, nitrogen dioxide; CO, Carbon monoxide; O_3_, ozone; ppb, parts per billion. ^a^Adjusted for age, monthly household income, education, drinking, smoking, physical activity, temperature, humidity. ^b^Adjusted for age, sex, monthly household income, education, drinking, smoking, physical activity, temperature, humidity. ^c^Adjusted for age, sex, monthly household income, education, drinking, physical activity, temperature, humidity

The results of the stratified analyses are presented in Fig. 2. When adjusted for potential confounders, males had higher risks of developing MetS than females for all air pollutants. In the case of age, 60–69 year old age group showing the highest incidence rate for all air pollutants. As for smoking status, the highest HR was found in the past smoking group for all air pollutants followed by current smoking group.

### Sensitivity analysis

We conducted a sensitivity analysis by adding region, and BMI to the main model, respectively. This sensitivity analysis allowed us to observe whether region act as a confounder and BMI as an intermediate (*n* = 7,428). Table S6 shows the results of sensitivity analysis. Model 3 shows the results of HR after adding region to the main model. The incidence rate increased with a HR of 1.29 (95% CI: 1.13-1.46) for PM_2.5_, 2.78 (95% CI: 2.48-3.12) for SO_2_, and 7.08 (95% CI: 5.54-9.03) for CO. Model 4, which shows the results of HR after adding BMI to the main model, the HR for SO_2_, NO_2_, and CO were 1.58 (95% CI: 1.47-1.69), 1.10 (95% CI: 1.02-1.19), and 1.61 (95% CI: 1.47-1.77) respectively. Model 4 showed similar results to those of the main analysis.

## Discussion

Using a large-scale population-based cohort, we observed a significant association between the annual concentration of ambient air pollution and the incidences of MetS after adjusting for potential confounders. Exposure to PM_2.5_, SO_2_, NO_2_, and CO were positively associated with higher risk of MetS. In a two-pollutant model performed between PM_2.5_ and NO_2_, SO_2_, O_3_, we observed statistical significance in PM_2.5_, and SO_2_. In the subgroup analysis, males had a higher risk of developing MetS than females for all air pollutants. In the case of age, the 60–69 year old age group had the highest HR for all air pollutants. We also found a significant association between air pollution exposure and the incidences of MetS components, with the highest HR shown in SO_2_ and CO for all of the components.

Depending on the pollutant, there are similarities and differences between the present and previous studies conducted. Positive associations were reported between long-term PM exposure and MetS incidence, however evidence for ozone exposure is highly mixed. Long-term PM exposure increased the MetS incidences, which was consistent with our results [13, 15, 22, 23]. A previous retrospective cohort study among health examinees in Korea showed 10 µg/m^3^ annual increase of PM_2.5_ significantly increases the MetS incidence by 7% [15]. A study conducted in the UK showed increase in the mean annual PM_2.5_ concentration by 1 µg/m^3^ was associated with a higher risk of MetS (HR: 1.27, 95% CI: 1.06-1.52) [16], and a study from China also showed similar results (HR: 1.027, 95% CI: 1.006-1.048) for every 10 µg/m^3^ increase in PM_10_ [13]. We also observed significant increases in MetS components, which were consistent with the results of previous studies conducted worldwide [39–42].

However, for ozone exposure, a negative association between O_3_ exposure and the incidences of MetS and its components was observed in the present study. This is inconsistent with some previous results. A study conducted among Taiwanese population has shown 3.30 ppb increase of ozone exposure increase the incidence of hyperglycemia by 5.8% [43]. 10 µg/m^3^ annual increase of ozone increased the incidence of diabetes with HR of 1.015 (95% CI: 1.992-1.027) in a study conducted among the Rome population [44]. The reason for this inconsistency may have been due to first, the association between O_3_ and other pollutants. O_3_ is a secondary substance produced by photochemical decomposition of nitrogen oxide (NO_x_) and volatile organic compounds (VOCs) [45, 46]. Due to this characteristic, O_3_ and other air pollutants are negatively correlated [47], and generally has a positive correlation with temperature. Second, the inconsistency among studies may have been due to ethnicity, cultural and environmental factors, differences in air pollutant composition and severity, and study design [43]. Third, because MetS is defined by the presence of three or more symptoms, this might have affected the sample size which can led to inconsistent results [48]. O_3_ is known to have a positive correlation with temperature, however we observed no correlation in this study. The reason for this inconsistency may be first, because we used the annual average concentration of air pollution and meteorological variables, fluctuation within a year may be largely irrelevant. Second, O_3_ concentration formed through not only by temperature, but also by other meteorological variables such as relative humidity, wind direction, stability, and Long-Range Transport (LRT) is also known to affect the O_3_ concentration [49]. LRT was proven to be a major factor affecting ozone concentrations in non-urban areas in Korea by previous studies [49–51]. The lack of a high correlation between O_3_ and temperature may be explained by the fact that the study regions in this study were mostly non-urban areas.

We adjusted for temperature and relative humidity to adjust the regional and climate effects of air pollutant exposure. We observed a negative correlation between temperature and air pollutants, which was the highest in CO in this study. For the relative humidity, a negative correlation between air pollutants except for NO_2_ and O_3_ was observed. This is a similar result to that of previous studies showing a negative correlation between air pollutants, temperature, and relative humidity except for O_3_ [50, 52–54]. The reason why the results contrasted with those of this study and previous studies was interpreted as the particles that make up air pollutants may vary by country, region, and period. The correlation coefficient between temperature and CO was the highest in the negative direction in the present study. This correlation between meteorological variables and air pollutants explains the reason that incidence rate of MetS increased the most in CO.

In the sensitivity analysis, we performed analyses by adding region in Model 3, and BMI in Model 4, to the main model respectively. After adding region in the statistical model, we observed a significant increase in HR in the results. The spatiotemporal characteristics of air pollutants are associated with local industries and environments. Also, geographically adjacent cities tend to have similar air pollutant concentrations and meteorological patterns [55]. We adjusted for temperature and relative humidity to adjust the regional and climate effects. Therefore, we did not further adjust for region in the main model, because doing so could lead to an overestimation. In addition, BMI may be a mediator but we did not observe a significant difference in HR with or without adjustment in this study, therefore, we concluded that BMI does not act as a mediator at least in our dataset, therefore we did not adjust for BMI in the present study.

In previous studies where two-pollutant model was performed analyzing the association between air pollution and MetS, PM_2.5_ and PM_10_ was found to be positively associated with MetS incidence [56, 57]. In the present study, we found PM_2.5_, and SO_2_ were positively associated with MetS incidence in two-pollutant analysis, showing similar robust results as the main analysis. In particular, SO_2_ was more strongly associated with the outcome in comparison to each individual compound. However, when adding O_3_ to the model with PM_2.5_ as the main pollutant, the results were not statistically significant nor robust to the main result. This is partly inconsistent with previous studies, and the reason for the inconsistency may be due to differences in environmental factors, air pollutant composition, study design, and sample size. Also, high correlation between air pollutants may under estimated the result of two-pollutant analysis.

Although there were not any interventions provided at follow-up surveys, such as recommendations on changing lifestyle patterns, we observed the highest HR in the past smoking group for all air pollutants in subgroup analysis. To the best of our knowledge, there were not any previous studies that observed the highest HR in the past smoking group. However, the reason for this may be because the effect of air pollution may be lower in current smokers due to the effects of smoking [58].

There is no clear basis for how the air pollutants cause MetS, but it has been shown in previous studies that they have a systematic effect on the body. When air pollutants are inhaled through the respiratory tract, they induce DNA methylation, oxidative stress, and pro-inflammatory cytokine secretion. This causes systematic inflammation and impairment in the autonomic nervous system, circulates throughout the body within the blood, and affects the entire human body. Due to this systematic effect, previous studies have shown that DNA methylation can induce epigenetic changes and increase the susceptibility of MetS [59]. For oxidative stress and inflammatory markers, studies have shown that they play an important role in the pathogenesis of metabolic syndrome. Reactive oxygen species (ROS) was proven to cause mitochondrial dysfunction, promote protein damage, trigger lipid peroxidation, and antioxidant defenses in metabolic syndrome in previous studies. Disrupted cell signaling pathways elevate inflammatory markers, lipid peroxides, and free radicals, leading to cellular damage and the clinical symptoms of metabolic syndrome [60]. According to previous studies, DNA methylation can be observed in as short as 5 to 30 moving-average days [61, 62], systematic inflammation and pro-inflammatory cytokine secretion in less than 1 year [63, 64], and oxidative stress in 7 moving-average days [65]. These mechanisms explain the incidence of hypertension, insulin resistance, abdominal obesity, and MetS [66–70].

Although we found a significant association between air pollution and MetS incidences, there were some potential limitations that should be noted when interpreting the results. First, individual exposure was estimated based on the participants’ residential addresses without considering actual exposure during daytime activities. Also, due to lack of information, participants’ addresses one year prior to enrollment were not considered. There might be misclassification of exposure due to these reasons, however, this misclassification is likely to be random and causes bias towards the null [71, 72]. Thus, the association between air pollutants and MetS may have been greater if we collected the actual exposure data. Second, two-pollutant analysis was only applied between PM_2.5_, and NO_2_, SO_2_, and O_3_. The air pollutants were highly correlated, and other pollutants need to be analyzed in two- or multi pollutant models to find out how independent pollutants affected the incidences of MetS. Due to the heterogeneity of the study areas, the composition of air pollutants and interaction between air pollutants may have varied [73, 74]. Third, although several important confounders were controlled, there may have been some other unmeasured covariates not considered. Fourth, the result of present study cannot be generalized to other race or ethnicity since the study participants only consisted of Korean. Fifth, we only considered average annual concentrations of air pollutants as exposure variable. Considering that exposure to the air pollutants in a long period would gradually induce the MetS, there probably exists a certain period which is critical to the MetS occurrence. However, the exact time point of occurrence of MetS cannot be determined and we think average concentration as exposure is appropriate approach. Sixth, the present study did not examine the non-linear association between air pollutants and MetS. Most of the previous studies did not assume nonlinearity between air pollutants exposure and MetS incidence [42, 75]. However, there is possibility of the true association being non-linear with threshold, and the present result may be under- or overestimate the true association in the concentration range higher and lower than the threshold, respectively.

## Conclusion

We found that long-term exposure to PM_2.5_, SO_2_, NO_2_, and CO significantly increased the incidences of MetS among Korean adults. Similar results were found for the components of MetS. In two-pollutant analysis, PM_2.5_, and SO_2_ showed positive association with MetS. In particular, the incidence rate tended to increase in the males and elderly population, so appropriate environmental regulations and healthcare for susceptible population is needed.

## Electronic supplementary material

Below is the link to the electronic supplementary material.

Supplementary Material 1

## Data Availability

No datasets were generated or analysed during the current study.

## Abbreviations

HR: Hazard ratio
95% CI: 95% confidence interval
IQR: Interquartile range
BMI: Body mass index
PM_2.5_: Particulate matter with aerodynamic diameters ≤ 2.5 μm
SO_2_: Sulfur dioxide
NO_2_: Nitrogen dioxide
CO: Carbon monoxide
O_3_: Ozone
ppb: Parts per billion
KoGES: Korean Genome and Epidemiology Study

## References

[1] James M, Varghese TP, Sharma R, Chand S. Association between metabolic syndrome and diabetes mellitus according to international diabetic federation and national cholesterol education program adult treatment panel III criteria: a cross-sectional study. J Diabetes Metab Disord. 2020;19(1):437–43. 10.1007/s40200-020-00523-2 32550195 10.1007/s40200-020-00523-2 PMC7270215

[2] Gustavo deS, Barbalho Y, Morato Stival M, Ramos de Lima L, Cristina Rodrigues da Silva I, de Oliveira Silva A, et al. Vieira Gomes da Costa M,. Impact of Metabolic Syndrome Components in High-Risk Cardiovascular Disease Development in Older Adults. Clin Interv Aging. 2020;15:1691– 700. 10.2147/cia.s252589 10.2147/CIA.S252589 PMC7513792 33061322

[3] Mottillo S, Filion KB, Genest J, Joseph L, Pilote L, Poirier P, et al. The metabolic syndrome and cardiovascular risk a systematic review and meta-analysis. J Am Coll Cardiol. 2010;56(14):1113–32. 10.1016/j.jacc.2010.05.034 20863953 10.1016/j.jacc.2010.05.034

[4] Noubiap JJ, Nansseu JR, Lontchi-Yimagou E, Nkeck JR, Nyaga UF, Ngouo AT, et al. Geographic distribution of metabolic syndrome and its components in the general adult population: a meta-analysis of global data from 28 million individuals. Diabetes Res Clin Pract. 2022;188:109924. 10.1016/j.diabres.2022.109924 35584716 10.1016/j.diabres.2022.109924

[5] Hirode G, Wong RJ. Trends in the prevalence of metabolic syndrome in the United States, 2011–2016. JAMA. 2020;323(24):2526–8. 10.1001/jama.2020.4501 32573660 10.1001/jama.2020.4501 PMC7312413

[6] World Health Organization. Air Pollution. 2021. https://www.who.int/health-topics/air-pollution#tab=tab_1 Accessed Jul 17 2024.

[7] United States Environmental Protection Agency. Hazardous Air Pollutants. 2023. https://www.epa.gov/haps/hazardous-air-pollutants-sources-and-exposure Accessed Jul 17 2024.

[8] Krall JR, Strickland MJ. Recent approaches to estimate associations between source-specific air pollution and health. Curr Environ Health Rep. 2017;4(1):68–78. 10.1007/s40572-017-0124-5 28108914 10.1007/s40572-017-0124-5

[9] GBD 2019 Risk Factors Collaborators. Global burden of 87 risk factors in 204 countries and territories, 1990–2019: a systematic analysis for the global burden of Disease Study 2019. Lancet. 2020;396(10258):1223–49. 33069327 10.1016/S0140-6736(20)30752-2 PMC7566194

[10] Sang S, Chu C, Zhang T, Chen H, Yang X. The global burden of disease attributable to ambient fine particulate matter in 204 countries and territories, 1990–2019: a systematic analysis of the global burden of Disease Study 2019. Ecotoxicol Environ Saf. 2022;238:113588. 10.1016/j.ecoenv.2022.113588 35525115 10.1016/j.ecoenv.2022.113588

[11] Rochlani Y, Pothineni NV, Kovelamudi S, Mehta JL. Metabolic syndrome: pathophysiology, management, and modulation by natural compounds. Ther Adv Cardiovasc Dis. 2017;11(8):215–25. 10.1177/1753944717711379 28639538 10.1177/1753944717711379 PMC5933580

[12] Cornier MA, Dabelea D, Hernandez TL, Lindstrom RC, Steig AJ, Stob NR, et al. The metabolic syndrome. Endocr Rev. 2008;29(7):777–822. 10.1210/er.2008-0024 18971485 10.1210/er.2008-0024 PMC5393149

[13] Liu F, Wang X, Pan M, Zhang K, Zhou F, Tong J, et al. Exposure to air pollution and prevalence of metabolic syndrome: a nationwide study in China from 2011 to 2015. Sci Total Environ. 2023;855:158596. 10.1016/j.scitotenv.2022.158596 36089046 10.1016/j.scitotenv.2022.158596

[14] Zang ST, Luan J, Li L, Wu QJ, Chang Q, Dai HX, et al. Air pollution and metabolic syndrome risk: evidence from nine observational studies. Environ Res. 2021;202:111546. 10.1016/j.envres.2021.111546 34265350 10.1016/j.envres.2021.111546

[15] Lee S, Park H, Kim S, Lee EK, Lee J, Hong YS, et al. Fine particulate matter and incidence of metabolic syndrome in non-CVD patients: a nationwide population-based cohort study. Int J Hyg Environ Health. 2019;222(3):533–40. 10.1016/j.ijheh.2019.01.010 30797734 10.1016/j.ijheh.2019.01.010

[16] Wallwork RS, Colicino E, Zhong J, Kloog I, Coull BA, Vokonas P, et al. Ambient fine particulate matter, Outdoor temperature, and risk of metabolic syndrome. Am J Epidemiol. 2017;185(1):30–9. 10.1093/aje/kww157 27927620 10.1093/aje/kww157 PMC5209587

[17] Wang C, Chen R, Cai J, Shi J, Yang C, Tse LA, et al. Personal exposure to fine particulate matter and blood pressure: a role of angiotensin converting enzyme and its DNA methylation. Environ Int. 2016;94:661–6. 10.1016/j.envint.2016.07.001 27397929 10.1016/j.envint.2016.07.001

[18] Kim JS, Chen Z, Alderete TL, Toledo-Corral C, Lurmann F, Berhane K, et al. Associations of air pollution, obesity and cardiometabolic health in young adults: the Meta-AIR study. Environ Int. 2019;133(Pt A):105180. 10.1016/j.envint.2019.105180 10.1016/j.envint.2019.105180 PMC6884139 31622905

[19] Xu MX, Ge CX, Qin YT, Gu TT, Lou DS, Li Q, et al. Prolonged PM_2.5_ exposure elevates risk of oxidative stress-driven nonalcoholic fatty liver disease by triggering increase of dyslipidemia. Free Radic Biol Med. 2019;130:542–56. 10.1016/j.freeradbiomed.2018.11.016 30465824 10.1016/j.freeradbiomed.2018.11.016

[20] Ning J, Zhang Y, Hu H, Hu W, Li L, Pang Y, et al. Association between ambient particulate matter exposure and metabolic syndrome risk: a systematic review and meta-analysis. Sci Total Environ. 2021;782:146855. 10.1016/j.scitotenv.2021.146855 33839664 10.1016/j.scitotenv.2021.146855

[21] Yang BY, Qian ZM, Li S, Fan S, Chen G, Syberg KM, et al. Long-term exposure to ambient air pollution (including PM_1_) and metabolic syndrome: the 33 communities Chinese Health Study (33CCHS). Environ Res. 2018;164:204–11. 10.1016/j.envres.2018.02.029 29501830 10.1016/j.envres.2018.02.029

[22] Chen YC, Chin WS, Pan SC, Wu CD, Guo YLL. Long-term exposure to Air Pollution and the occurrence of metabolic syndrome and its components in Taiwan. Environ Health Perspect. 2023;131(1):17001. 10.1289/ehp10611 36598238 10.1289/EHP10611 PMC9811992

[23] Yang BY, Bloom MS, Markevych I, Qian ZM, Vaughn MG, Cummings-Vaughn LA, et al. Exposure to ambient air pollution and blood lipids in adults: the 33 communities Chinese Health Study. Environ Int. 2018;119:485–92. 10.1016/j.envint.2018.07.016 30048882 10.1016/j.envint.2018.07.016

[24] Hwang MJ, Kim JH, Koo YS, Yun HY, Cheong HK. Impacts of ambient air pollution on glucose metabolism in Korean adults: a Korea national health and nutrition examination survey study. Environ Health. 2020;19(1):70. 10.1186/s12940-020-00623-9 32552747 10.1186/s12940-020-00623-9 PMC7302244

[25] Shin J, Choi J, Kim KJ. Association between long-term exposure of ambient air pollutants and cardiometabolic diseases: a 2012 Korean Community Health Survey. Nutr Metab Cardiovasc Dis. 2019;29(2):144–51. 10.1016/j.numecd.2018.09.008 30595346 10.1016/j.numecd.2018.09.008

[26] Adar SD, Chen YH, D’Souza JC, O’Neill MS, Szpiro AA, Auchincloss AH, et al. Longitudinal analysis of long-term air pollution levels and blood pressure: a cautionary tale from the multi-ethnic study of atherosclerosis. Environ Health Perspect. 2018;126(10):107003. 10.1289/ehp2966 30392401 10.1289/EHP2966 PMC6371645

[27] Eze IC, Schaffner E, Foraster M, Imboden M, von Eckardstein A, Gerbase MW, et al. Long-term exposure to ambient air pollution and metabolic syndrome in adults. PLoS ONE. 2015;10(6):e0130337. 10.1371/journal.pone.0130337 26103580 10.1371/journal.pone.0130337 PMC4478007

[28] Qin P, Luo X, Zeng Y, Zhang Y, Li Y, Wu Y, et al. Long-term association of ambient air pollution and hypertension in adults and in children: a systematic review and meta-analysis. Sci Total Environ. 2021;796:148620. 10.1016/j.scitotenv.2021.148620 34274662 10.1016/j.scitotenv.2021.148620

[29] Yang BY, Fan S, Thiering E, Seissler J, Nowak D, Dong GH, et al. Ambient air pollution and diabetes: a systematic review and meta-analysis. Environ Res. 2020;180:108817. 10.1016/j.envres.2019.108817 31627156 10.1016/j.envres.2019.108817

[30] Yu L, Liu W, Wang X, Ye Z, Tan Q, Qiu W, et al. A review of practical statistical methods used in epidemiological studies to estimate the health effects of multi-pollutant mixture. Environ Pollut. 2022;306:119356. 10.1016/j.envpol.2022.119356 35487468 10.1016/j.envpol.2022.119356

[31] National Institute of Health. Korean Genome and Epidemiology Study. 2023. https://nih.go.kr/ko/main/contents.do?menuNo=300563 Accessed Jul 17 2024.

[32] Kim Y, Han BG, KoGES group. Cohort Profile: the Korean Genome and Epidemiology Study (KoGES) Consortium. Int J Epidemiol. 2017;46(2):e20. 10.1093/ije/dyv316 27085081 10.1093/ije/dyv316 PMC5837648

[33] Ryou HG, Heo J, Kim SY. Source apportionment of PM_10_ and PM_2.5_ air pollution, and possible impacts of study characteristics in South Korea. Environ Pollut. 2018;240:963–72. 10.1016/j.envpol.2018.03.066 29910064 10.1016/j.envpol.2018.03.066

[34] Woo HD, Song DS, Choi SH, Park JK, Lee K, Yun HY, et al. Integrated dataset of the Korean genome and epidemiology study cohort with estimated air pollution data. Epidemiol Health. 2022;44:e2022071. 10.4178/epih.e2022071 36108673 10.4178/epih.e2022071 PMC9849844

[35] Alberti KGMM, Eckel RH, Grundy SM, Zimmet PZ, Cleeman JI, Donato KA, et al. Harmonizing the metabolic syndrome: a joint interim statement of the international diabetes federation task force on epidemiology and prevention; National Heart, Lung, and Blood Institute; American Heart Association; World Heart Federation; International Atherosclerosis Society; and International Association for the Study of Obesity. Circulation. 2009;120(16):1640–5. 10.1161/circulationaha.109.192644 19805654 10.1161/CIRCULATIONAHA.109.192644

[36] World Health Organization, Regional Office for the Western Pacific. The Asia-Pacific perspective: redefining obesity and its treatment. Sydney: Health Communications Australia; 2000. p. 55. https://iris.who.int/handle/10665/206936

[37] Textor J, Hardt J, Knüppel S. DAGitty: a graphical tool for analyzing causal diagrams. Epidemiology. 2011;22(5):745. 10.1097/ede.0b013e318225c2be 10.1097/EDE.0b013e318225c2be 21811114

[38] World Health Organization. WHO global air quality guidelines. Particulate matter (PM2.5 and PM10), ozone, nitrogen dioxide, sulfur dioxide and carbon monoxide. 2021. https://www.who.int/publications/i/item/9789240034228 Accessed Jul 17 2024. 34662007

[39] Shin MK, Kim KN. Association between long-term air pollution exposure and development of diabetes among community-dwelling adults: modification of the associations by dietary nutrients. Environ Int. 2023;174:107908. 10.1016/j.envint.2023.107908 37004480 10.1016/j.envint.2023.107908

[40] Shamy M, Alghamdi M, Khoder MI, Mohorjy AM, Alkhatim AA, Alkhalaf AK, et al. Association between exposure to Ambient Air Particulates and metabolic Syndrome Components in a Saudi Arabian Population. Int J Environ Res Public Health. 2017;15(1):27. 10.3390/ijerph15010027 29295575 10.3390/ijerph15010027 PMC5800127

[41] Coogan PF, White LF, Yu J, Burnett RT, Seto E, Brook RD, et al. PM_2.5_ and diabetes and hypertension incidence in the Black women’s Health Study. Epidemiology. 2016;27(2):202–10. 10.1097/ede.0000000000000418 26595125 10.1097/EDE.0000000000000418 PMC5499991

[42] Yitshak Sade M, Shi L, Colicino E, Amini H, Schwartz JD, Di Q, et al. Long-term air pollution exposure and diabetes risk in American older adults: a national secondary data-based cohort study. Environ Pollut. 2023;320:121056. 10.1016/j.envpol.2023.121056 36634862 10.1016/j.envpol.2023.121056 PMC9905312

[43] Li YL, Chuang TW, Chang PY, Lin LY, Su CT, Chien LN, et al. Long-term exposure to ozone and sulfur dioxide increases the incidence of type 2 diabetes mellitus among aged 30 to 50 adult population. Environ Res. 2021;194:110624. 10.1016/j.envres.2020.110624 33412098 10.1016/j.envres.2020.110624

[44] Renzi M, Cerza F, Gariazzo C, Agabiti N, Cascini S, Di Domenicantonio R, et al. Air pollution and occurrence of type 2 diabetes in a large cohort study. Environ Int. 2018;112:68–76. 10.1016/j.envint.2017.12.007 29253730 10.1016/j.envint.2017.12.007

[45] Hoffmann B, Luttmann-Gibson H, Cohen A, Zanobetti A, de Souza C, Foley C, et al. Opposing effects of particle pollution, ozone, and ambient temperature on arterial blood pressure. Environ Health Perspect. 2012;120(2):241–6. 10.1289/ehp.1103647 22020729 10.1289/ehp.1103647 PMC3279434

[46] Jia MW, Zhao TL, Cheng XH, Gong SL, Zhang XZ, Tang LL, et al. Inverse relations of PM_2.5_ and O_3_ in air compound Pollution between Cold and Hot Seasons over an urban area of East China. Atmosphere. 2017;8(3):59. 10.3390/atmos8030059

[47] Chen J, Shen H, Li T, Peng X, Cheng H, Ma AC. Temporal and spatial features of the correlation between PM_2.5_ and O_3_ concentrations in China. Int J Environ Res Public Health. 2019;16(23):4824. 10.3390/ijerph16234824 31801295 10.3390/ijerph16234824 PMC6926570

[48] LaKind JS, Burns CJ, Pottenger LH, Naiman DQ, Goodman JE, Marchitti SA. Does ozone inhalation cause adverse metabolic effects in humans? A systematic review. Crit Rev Toxicol. 2021;51(6):467–508. 10.1080/10408444.2021.1965086 34569909 10.1080/10408444.2021.1965086

[49] Shin HJ, Park JH, Park JS, Song IH, Park SM, Roh SA, et al. The Long Term trends of Tropospheric ozone in Major regions in Korea. Asian J Atmospheric Environ. 2017;11(4):235–53. 10.5572/ajae.2017.11.4.235

[50] Allabakash S, Lim S, Chong KS, Yamada TJ. Particulate matter concentrations over South Korea: impact of Meteorology and other pollutants. Remote Sens. 2022;14(19):4849. 10.3390/rs14194849

[51] Yeo MJ, Kim YP. Long-term trends of surface ozone in Korea. J Clean Prod. 2021;294:125352. 10.1016/j.jclepro.2020.125352

[52] Fu W, Chen Z, Zhu Z, Liu Q, van den Bosch CCK, Qi J, et al. Spatial and temporal variations of six Criteria Air pollutants in Fujian Province, China. Int J Environ Res Public Health. 2018;15(12):2846. 10.3390/ijerph15122846 30551634 10.3390/ijerph15122846 PMC6313486

[53] Tai APK, Mickley LJ, Jacob DJ. Correlations between fine particulate matter (PM_2.5_) and meteorological variables in the United States: implications for the sensitivity of PM_2.5_ to climate change. Atmos Environ. 2010;44(32):3976–84. 10.1016/j.atmosenv.2010.06.060

[54] Yang Q, Yuan Q, Li T, Shen H, Zhang L. The relationships between PM_2.5_ and Meteorological factors in China: Seasonal and Regional variations. Int J Environ Res Public Health. 2017;14(12):1510. 10.3390/ijerph14121510 29206181 10.3390/ijerph14121510 PMC5750928

[55] Chen Z, Chen D, Zhao C, Kwan MP, Cai J, Zhuang Y, et al. Influence of meteorological conditions on PM(2.5) concentrations across China: a review of methodology and mechanism. Environ Int. 2020;139:105558. 10.1016/j.envint.2020.105558 32278201 10.1016/j.envint.2020.105558

[56] Feng S, Meng Q, Guo B, Guo Y, Chen G, Pan Y, et al. Joint exposure to air pollution, ambient temperature and residential greenness and their association with metabolic syndrome (MetS): a large population-based study among Chinese adults. Environ Res. 2022;214(Pt 1):113699. 10.1016/j.envres.2022.113699 35714687 10.1016/j.envres.2022.113699

[57] Voss S, Schneider A, Huth C, Wolf K, Markevych I, Schwettmann L, et al. Long-term exposure to air pollution, road traffic noise, residential greenness, and prevalent and incident metabolic syndrome: results from the population-based KORA F4/FF4 cohort in Augsburg, Germany. Environ Int. 2021;147:106364. 10.1016/j.envint.2020.106364 33421766 10.1016/j.envint.2020.106364

[58] Gao W, Sanna M, Hefler M, Wen CP. Air pollution is not ‘the new smoking’: comparing the disease burden of air pollution and smoking across the globe, 1990–2017. Tob Control. 2020;29(6):715–8. 10.1136/tobaccocontrol-2019-055181 31611424 10.1136/tobaccocontrol-2019-055181 PMC7591797

[59] Poursafa P, Kamali Z, Fraszczyk E, Boezen HM, Vaez A, Snieder H. DNA methylation: a potential mediator between air pollution and metabolic syndrome. Clin Epigenetics. 2022;14(1):82. 10.1186/s13148-022-01301-y 35773726 10.1186/s13148-022-01301-y PMC9245491

[60] Masenga SK, Kabwe LS, Chakulya M, Kirabo A. Mechanisms of oxidative stress in metabolic syndrome. Int J Mol Sci. 2023;24(9). 10.3390/ijms24097898 10.3390/ijms24097898 PMC10178199 37175603

[61] De Prins S, Koppen G, Jacobs G, Dons E, Van de Mieroop E, Nelen V, et al. Influence of ambient air pollution on global DNA methylation in healthy adults: a seasonal follow-up. Environ Int. 2013;59:418–24. 10.1016/j.envint.2013.07.007 23917442 10.1016/j.envint.2013.07.007

[62] Plusquin M, Guida F, Polidoro S, Vermeulen R, Raaschou-Nielsen O, Campanella G, et al. DNA methylation and exposure to ambient air pollution in two prospective cohorts. Environ Int. 2017;108:127–36. 10.1016/j.envint.2017.08.006 28843141 10.1016/j.envint.2017.08.006 PMC6139298

[63] Klümper C, Krämer U, Lehmann I, von Berg A, Berdel D, Herberth G, et al. Air pollution and cytokine responsiveness in asthmatic and non-asthmatic children. Environ Res. 2015;138:381–90. 10.1016/j.envres.2015.02.034 25769127 10.1016/j.envres.2015.02.034

[64] Tripathy S, Marsland AL, Kinnee EJ, Tunno BJ, Manuck SB, Gianaros PJ, et al. Long-term Ambient Air Pollution exposures and circulating and stimulated inflammatory mediators in a cohort of midlife adults. Environ Health Perspect. 2021;129(5):57007. 10.1289/ehp7089 34014775 10.1289/EHP7089 PMC8136520

[65] Li W, Wilker EH, Dorans KS, Rice MB, Schwartz J, Coull BA, et al. Short-term exposure to air pollution and biomarkers of oxidative stress: the Framingham heart study. J Am Heart Assoc. 2016;5(5):e002742. 10.1161/jaha.115.002742 27126478 10.1161/JAHA.115.002742 PMC4889166

[66] Brook RD, Rajagopalan S. Particulate matter, air pollution, and blood pressure. J Am Soc Hypertens. 2009;3(5):332–50. 10.1016/j.jash.2009.08.005 20409976 10.1016/j.jash.2009.08.005

[67] Brook RD, Sun Z, Brook JR, Zhao X, Ruan Y, Yan J, et al. Extreme air pollution conditions adversely affect blood pressure and insulin resistance: the air pollution and cardiometabolic disease study. Hypertension. 2016;67(1):77–85. 10.1161/HYPERTENSIONAHA.115.06237 26573709 10.1161/HYPERTENSIONAHA.115.06237 PMC4830086

[68] Clementi EA, Talusan A, Vaidyanathan S, Veerappan A, Mikhail M, Ostrofsky D, et al. Metabolic syndrome and air pollution: a narrative review of their cardiopulmonary effects. Toxics. 2019;7(1):6. 10.3390/toxics7010006 30704059 10.3390/toxics7010006 PMC6468691

[69] Münzel T, Gori T, Al-Kindi S, Deanfield J, Lelieveld J, Daiber A, et al. Effects of gaseous and solid constituents of air pollution on endothelial function. Eur Heart J. 2018;39(38):3543–50. 10.1093/eurheartj/ehy481 30124840 10.1093/eurheartj/ehy481 PMC6174028

[70] Rajagopalan S, Brook RD. Air pollution and type 2 diabetes: mechanistic insights. Diabetes. 2012;61(12):3037–45. 10.2337/db12-0190 23172950 10.2337/db12-0190 PMC3501850

[71] Hutcheon JA, Chiolero A, Hanley JA. Random measurement error and regression dilution bias. BMJ. 2010;340:c2289. 10.1136/bmj.c2289 20573762 10.1136/bmj.c2289

[72] Jun YB, Song I, Kim OJ, Kim SY. Impact of limited residential address on health effect analysis of predicted air pollution in a simulation study. J Expo Sci Environ Epidemiol. 2022;32(4):637–43. 10.1038/s41370-022-00412-1 35082387 10.1038/s41370-022-00412-1 PMC9349037

[73] Traini E, Huss A, Portengen L, Rookus M, Verschuren WMM, Vermeulen RCH, et al. A multipollutant approach to estimating causal effects of air pollution mixtures on overall mortality in a large, prospective cohort. Epidemiology. 2022;33(4):514–22. 10.1097/ede.0000000000001492 35384897 10.1097/EDE.0000000000001492 PMC9148665

[74] Su PF, Sie FC, Yang CT, Mau YL, Kuo S, Ou HT. Association of ambient air pollution with cardiovascular disease risks in people with type 2 diabetes: a bayesian spatial survival analysis. Environ Health. 2020;19(1):110. 10.1186/s12940-020-00664-0 33153466 10.1186/s12940-020-00664-0 PMC7643356

[75] Zhang JS, Gui ZH, Zou ZY, Yang BY, Ma J, Jing J, et al. Long-term exposure to ambient air pollution and metabolic syndrome in children and adolescents: a national cross-sectional study in China. Environ Int. 2021;148:106383. 10.1016/j.envint.2021.106383 33465664 10.1016/j.envint.2021.106383

