# Supplementary material for: Association between annual concentration of air pollutants and incidence of metabolic syndrome among Korean adults: Korean Genome and Epidemiology Study (KoGES)
Source: Environ Health. 2025 Feb 11;24:3. doi: 10.1186/s12940-025-01158-7 (PMC11818349; doi:10.1186/s12940-025-01158-7)

Supplementary Material to

**Association between annual concentration of air pollutants and incidence of metabolic syndrome among Korean adults: Korean Genome and Epidemiology Study (KoGES)**

Hanuel Shin^1,2^, Minkyo Song^3^, Sanghyuk Bae^4^

^1^Graduate School of Public Health and Healthcare Management, The Catholic University of Korea, Seoul, Republic of Korea

^2^Department of Nursing, Seoul St. Mary’s Hospital, College of Medicine, The Catholic University of Korea, Seoul, Republic of Korea

^3^Immunoepidemiology Unit, Laboratory of Epidemiology and Population Sciences, National Institute on Aging, National Institutes of Health, USA

^4^Department of Preventive Medicine, College of Medicine, The Catholic University of Korea, Seoul, Republic of Korea

Table of contents

Table S1. Comparison of baseline descriptive characteristics of withdrawal and included population …………. 3

Table S2. Baseline descriptive characteristics of MetS and Non-MetS population ……………………………... 6

Table S3. Distribution of MetS and Non-MetS cases by each air pollutant variable by quartiles ………………. 9

Table S4. Number of participants by year of MetS occurence by region during follow-up …………………….. 10

Table S5. Hazard ratios of metabolic syndrome per interquartile range width increment in air pollution exposure during follow-up from different models ……………………………………………………………………….... 11

Table S6. Hazard ratios of metabolic syndrome per interquartile range with increment in air pollution exposure during follow-up using different covariates …………………………………………………………………….. 12

Figure S1. Geographical location of study regions ………………………………………………………….….. 13

Figure S2. Timeline of recruitment and exposure calculation ...………………………………………………... 14

Figure S3. Directed Acyclic graph (DAG) demonstrating the assumed causal relationships between the exposure, outcome, and other covariates ………………………………………………………………….……………….. 15

Figure S4. Spearman correlation coefficients of air pollutants exposure during follow-up ……………………. 16

Table S1 Comparison of baseline descriptive characteristics of withdrawal and included population (2005-2012)

| Characteristics | Included  (n= 7,428) | Withdrawal (n=20,909) | *P*-value |
| --- | --- | --- | --- |
| Age, year, mean ± SD | 58.2 ± 9.6 | 58.7 ± 9.3 | <0.01 |
| Sex, n (%) |  |  | <0.01 |
| Male | 3804 (51.2) | 7017 (33.6) |  |
| Female | 3624 (48.8) | 13892 (66.4) |  |
| Residence, n (%) |  |  | <0.01 |
| Yangpyeong | 1411 (17.7) | 2252 (10.8) |  |
| Namwon | 1109 (14.9) | 2865 (13.7) |  |
| Goryeong | 1215 (16.4) | 2347 (11.2) |  |
| Wonju | 1278 (17.2) | 2339 (11.20) |  |
| Pyeongchang | 1029 (13.9) | 1751 (8.4) |  |
| Ganghwa | 1486 (20.0) | 2732 (13.0) |  |
| Other | 0 | 6623 (31.7) |  |
| Monthly household income, KRW, n (%) |  |  | <0.01 |
| <1,000,000 | 1260 (17.0) | 6899 (33.0) |  |
| 1,000,000~1,990,000 | 1005 (13.5) | 9937 (47.5) |  |
| 2,000,000~3,990,000 | 1030 (13.9) | 2681 (12.8) |  |
| ≥4,000,000 | 304 (4.1) | 1228 (5.9) |  |
| Unknown | 3829 (51.6) | 164 (0.8) |  |
| Job status, n (%) |  |  | 0.052 |
| Yes | 6528 (87.9) | 20127 (96.3) |  |
| No | 287 (3.9) | 717 (3.4) |  |
| Unknown | 613 (8.3) | 65 (0.3) |  |
| Education, n (%) |  |  | <0.01 |
| Elementary school | 3585 (48.3) | 12694 (60.7) |  |
| Middle school | 1382 (18.6) | 3552 (17.0) |  |
| High school | 1697 (22.9) | 3692 (17.7) |  |
| Over college | 747 (10.1) | 802 (3.8) |  |
| Unknown | 17 (0.2) | 169 (0.8) |  |
| Drinking status, n (%) |  |  | <0.01 |
| Current | 3482 (46.9) | 8347 (39.9) |  |
| Past | 485 (6.5) | 1541 (7.4) |  |
| Never | 3454 (46.5) | 11021 (52.7) |  |
| Unknown | 7 (0.1) | 0 |  |
| Smoking status, n (%) |  |  | <0.01 |
| Current | 1112 (15.0) | 6314 (30.2) |  |
| Past | 1286 (17.3) | 2165 (10.4) |  |
| Never | 3863 (52.0) | 12430 (59.4) |  |
| Unknown | 1167 (15.7) | 0 |  |
| Physical activity, n (%) |  |  | 0.011 |
| 1-2 times a week | 565 (7.6) | 1376 (6.6) |  |
| 3-6 times a week | 1080 (14.5) | 2498 (11.9) |  |
| Everyday | 868 (11.7) | 2452 (11.7) |  |
| No | 4890 (65.8) | 14523 (69.5) |  |
| Unknown | 25 (0.3) | 60 (0.3) |  |
| BMI, n (%) |  |  | <0.01 |
| < 18.5kg/m² | 184 (2.5) | 412 (2.0) |  |
| 18.5-22.9kg/m² | 2915 (39.2) | 6103 (29.2) |  |
| 23.0-24.9kg/m² | 1991 (26.8) | 5286 (25.3) |  |
| 25.0-29.9kg/m² | 2338 (31.5) | 9108 (43.6) |  |
| ≥30kg/m² | 0 | 0 |  |
| Family history of Hypertension, n (%) |  |  | 0.034 |
| Yes | 1518 (20.4) | 4216 (20.2) |  |
| No | 5855 (78.8) | 16466 (78.8) |  |
| Unknown | 55 (0.7) | 227 (1.1) |  |
| Family history of Hyperglycemia, n (%) |  |  | <0.01 |
| Yes | 934 (12.6) | 2697 (12.9) |  |
| No | 6443 (86.8) | 17987 (86.0) |  |
| Unknown | 51 (0.7) | 225 (1.1) |  |
| Waist circumference, cm, mean ± SD | 82.7 ± 8.8 | 83.2 ± 13.5 | <0.01 |
| Systolic blood pressure, mmHg, mean ± SD | 121.5 ± 16.5 | 127.7 ± 19.1 | <0.01 |
| Diastolic blood pressure, mmHg, mean ± SD | 77.3 ±10.5 | 80.0 ± 11.9 | <0.01 |
| Fasting blood glucose, mg/dL, mean ± SD | 94.7 ± 18.9 | 100.0 ± 5.8 | <0.01 |
| Total cholesterol, mg/dL, mean ± SD | 195.4 ± 34.9 | 201.1 ± 8.4 | <0.01 |
| Triglyceride, mg/dL, mean ± SD | 123.1 ± 74.3 | 159.2 ± 106.5 | <0.01 |
| High density lipoprotein cholesterol, mg/dL, mean ± SD | 46.6 ± 11.4 | 44.9 ± 11.1 | <0.01 |
| PM_2.5,_ µg/m^3^, mean ± SD | 27.50 ± 3.06 | 26.32 ± 3.27 | <0.01 |
| SO_2,_ ppb, mean ± SD | 4.68 ± 1.09 | 4.52 ± 1.33 | <0.01 |
| NO_2_, ppb, mean ± SD | 15.66 ± 5.18 | 15.29 ± 4.93 | <0.01 |
| CO, ppb, mean ± SD | 525.86 ± 113.75 | 516.27 ± 108.51 | <0.01 |
| O_3_, ppb, mean ± SD | 25.02 ± 3.37 | 25.68 ± 4.18 | <0.01 |

Note: Data were shown as mean ± SD for continuous variables and number (%) for categorical variables.

Abbreviations: SD, Standard deviation; KRW, Korean won; BMI, Body mass index; PM_2.5_, particulate matter with aerodynamic diameters ≤ 2.5 μm; SO_2_, sulfur dioxide; NO_2_, nitrogen dioxide; CO, carbon monoxide; O_3_, ozone; ppb, parts per billion.

Table S2 Baseline descriptive characteristics of MetS and Non-MetS population (2005-2012)

| Characteristics | Non-MetS (n= 5,655) | MetS (n= 1,773) | *P*-value |
| --- | --- | --- | --- |
| Age, year, mean ± SD | 58.1 ± 9.7 | 58.6 ± 9.0 | 0.079 |
| Sex, n (%) |  |  | <0.01 |
| Male | 3046 (53.9) | 758 (42.8) |  |
| Female | 2609 (46.1) | 1015 (57.2) |  |
| Residence, n (%) |  |  | <0.01 |
| Yangpyeong | 1019 (18.0) | 292 (16.5) |  |
| Namwon | 804 (14.2) | 305 (17.2) |  |
| Goryeong | 903 (16.0) | 312 (17.6) |  |
| Wonju | 915 (16.2) | 363 (20.5) |  |
| Pyeongchang | 781 (13.8) | 248 (14.0) |  |
| Ganghwa | 1233 (21.8) | 253 (14.3) |  |
| Monthly household income, KRW, n (%) |  |  | 0.013 |
| <1,000,000 | 972 (17.2) | 288 (16.2) |  |
| 1,000,000~1,990,000 | 772 (13.7) | 233 (13.1) |  |
| 2,000,000~3,990,000 | 821 (14.5) | 20.9 (11.8) |  |
| ≥4,000,000 | 231 (4.1) | 73 (4.10) |  |
| Unknown | 2859 (50.6) | 970 (54.7) |  |
| Job status, n (%) |  |  | 0.682 |
| Yes | 4980 (88.1) | 1548 (87.3) |  |
| No | 214 (3.8) | 73 (4.1) |  |
| Unknown | 461 (8.2) | 152 (8.6) |  |
| Education, n (%) |  |  | <0.01 |
| Elementary school | 2660 (47.0) | 925 (52.2) |  |
| Middle school | 1059 (18.7) | 323 (18.2) |  |
| High school | 1347 (23.8) | 350 (19.7) |  |
| Over college | 578 (10.2) | 169 (9.5) |  |
| Unknown | 11 (0.2) | 6 (0.3) |  |
| Drinking status, n (%) |  |  | <0.01 |
| Current | 2666 (47.1) | 816 (46.0) |  |
| Past | 393 (6.9) | 92 (5.2) |  |
| Never | 2589 (45.8) | 865 (48.8) |  |
| Unknown | 7 (0.1) | 0 (0.0) |  |
| Smoking status, n (%) |  |  | <0.01 |
| Current | 864 (15.3) | 248 (14.0) |  |
| Past | 1003 (17.7) | 283 (16.0) |  |
| Never | 2807 (49.6) | 1056 (59.6) |  |
| Unknown | 981 (17.3) | 186 (10.5) |  |
| Physical activity, n (%) |  |  | 0.476 |
| 1-2 times a week | 424 (7.5) | 141 (8.0) |  |
| 3-6 times a week | 805 (14.2) | 275 (15.5) |  |
| Everyday | 668 (11.8) | 200 (11.3) |  |
| No | 3739 (66.1) | 1151 (64.9) |  |
| Unknown | 19 (0.3) | 6 (0.3) |  |
| BMI, n (%) |  |  | <0.01 |
| < 18.5kg/m² | 171 (3.0) | 13 (0.7) |  |
| 18.5-22.9kg/m² | 2410 (42.6) | 505 (28.5) |  |
| 23.0-24.9kg/m² | 1499 (26.5) | 492 (27.7) |  |
| 25.0-29.9kg/m² | 1575 (27.9) | 763 (43.0) |  |
| ≥30kg/m² | 0 | 0 |  |
| Family history of Hypertension, n (%) |  |  | 0.061 |
| Yes | 1124 (19.9) | 394 (22.2) |  |
| No | 4492 (79.4) | 1363 (76.9) |  |
| Unknown | 39 (0.7) | 16 (0.9) |  |
| Family history of Hyperglycemia, n (%) |  |  | <0.01 |
| Yes | 659 (11.7) | 275 (15.5) |  |
| No | 4958 (87.7) | 1485 (83.8) |  |
| Unknown | 38 (0.7) | 13 (0.7) |  |
| Waist circumference, cm, mean ± SD | 82.1 ± 8.8 | 84.5 ± 8.4 | <0.01 |
| Systolic blood pressure, mmHg, mean ± SD | 120.5 ± 16.4 | 124.7 ± 16.3 | <0.01 |
| Diastolic blood pressure, mmHg, mean ± SD | 76.8 ± 10.5 | 79.0 ± 10.5 | <0.01 |
| Fasting blood glucose, mg/dL, mean ± SD | 93.9 ± 17.6 | 97.3 ± 22.5 | <0.01 |
| Total cholesterol, mg/dL, mean ± SD | 194.6 ± 34.8 | 197.8 ± 35.0 | <0.01 |
| Triglyceride, mg/dL, mean ± SD | 117.8 ± 69.0 | 140.3 ± 87.2 | <0.01 |
| High density lipoprotein cholesterol, mg/dL, mean ± SD | 47.6 ± 11.7 | 43.6 ± 9.8 | <0.01 |

Note: Data were shown as mean ± SD for continuous variables and number (%) for categorical variables.

Abbreviations: SD, Standard deviation; KRW, Korean won; BMI, Body mass index.

Table S3 Distribution of MetS and Non-MetS cases by each air pollutant variable by quartiles

| Variables | Non-MetS  (n= 5,655) | MetS  (n= 1,773) |
| --- | --- | --- |
| PM_2.5_ (µg/m^3^), n (%) |  |  |
| Q1: 17.91-25.12 | 1441 (25.5) | 416 (23.5) |
| Q2: 25.13-26.59 | 1471 (26.0) | 386 (21.8) |
| Q3: 26.60-28.60 | 1364 (24.1) | 493 (27.8) |
| Q4: 28.61-35.67 | 1379 (24.4) | 478 (27.0) |
| SO_2_ (ppb), n (%) |  |  |
| Q1: 2.34-3.73 | 1441 (25.5) | 416 (23.5) |
| Q2: 3.74-4.30 | 1477 (26.1) | 380 (21.4) |
| Q3: 4.31-4.89 | 1463 (25.9) | 394 (22.2) |
| Q4: 4.90-8.75 | 1274 (22.5) | 583 (30.3) |
| NO_2_ (ppb), n (%) |  |  |
| Q1: 4.98-10.84 | 1433 (25.3) | 424 (23.9) |
| Q2: 10.85-15.05 | 1446 (25.6) | 411 (23.2) |
| Q3: 15.06-19.71 | 1440 (25.5) | 417 (23.5) |
| Q4: 19.72-36.77 | 1336 (23.6) | 521 (29.4) |
| CO (ppb), n (%) |  |  |
| Q1: 288.35-399.89 | 1467 (25.9) | 390 (22.0) |
| Q2: 399.90-468.00 | 1444 (25.5) | 413 (23.3) |
| Q3: 468.01-572.60 | 1439 (25.4) | 418 (23.6) |
| Q4: 572.61-725.73 | 1305 (23.1) | 552 (31.1) |
| O_3_ (ppb), n (%) |  |  |
| Q1: 18.04-23.77 | 1308 (23.1) | 549 (31.0) |
| Q2: 23.78-25.09 | 1374 (24.3) | 483 (27.2) |
| Q3: 25.10-26.42 | 1435 (25.4) | 422 (23.8) |
| Q4: 26.43-37.36 | 1538 (27.2) | 319 (18.0) |

Abbreviations: PM_2.5_, particulate matter with aerodynamic diameters ≤ 2.5 μm; SO_2_, sulfur dioxide; NO_2_, nitrogen dioxide; CO, carbon monoxide; O_3_, ozone; ppb, parts per billion. *P*-values for interaction were P <0.01.

Table S4 Number of participants by year of MetS occurrence by region during follow-up (n=1,773)

|  | 2007 | 2008 | 2009 | 2010 | 2011 | 2012 | 2013 | 2014 | 2015 | 2016 | Total |
| --- | --- | --- | --- | --- | --- | --- | --- | --- | --- | --- | --- |
| Yangpyeong | 31 | 47 | 31 | 35 | 46 | 27 | 21 | 11 | 22 | 21 | 292 |
| Namwon | 12 | 72 | 50 | 28 | 35 | 25 | 20 | 20 | 25 | 18 | 305 |
| Goryeong | 16 | 97 | 62 | 22 | 26 | 11 | 29 | 10 | 28 | 11 | 312 |
| Wonju | 1 | 71 | 39 | 40 | 43 | 39 | 54 | 28 | 20 | 28 | 363 |
| Pyeongchang | 2 | 58 | 7 | 22 | 59 | 29 | 26 | 13 | 24 | 8 | 248 |
| Ganghwa | 16 | 70 | 16 | 7 | 44 | 21 | 25 | 11 | 22 | 21 | 253 |

Table S5 Hazard ratios of metabolic syndrome per interquartile range width increment in air pollution exposure during follow-up from different models (n=7,428)

| Approach | Pollutant | HR (95% CI) |
| --- | --- | --- |
| Single-pollutant | PM_2.5_ | 1.19 (1.12-1.27) |
| Two-pollutant | PM_2.5_ | 0.63 (0.57-0.68) |
|  | O_3_ | 0.33 (0.29-0.36) |
| Two-pollutant | PM_2.5_ | 0.86 (0.79-0.93) |
|  | SO_2_ | 1.75 (1.60-1.91) |
| Two-pollutant | PM_2.5_ | 1.27 (1.16-1.39) |
|  | NO_2_ | 0.90 (0.80-1.01) |

Note: IQR for PM_2.5_: 3.48㎍/㎥, SO_2_: 1.17ppb, NO_2_: 8.87ppb, O_3_: 2.65ppb.

Abbreviations: HR, Hazard ratio; 95% CI, 95% confidence interval; PM_2.5_, particulate matter with aerodynamic diameters ≤ 2.5 μm; SO_2_, sulfur dioxide; NO_2_, nitrogen dioxide; CO, carbon monoxide; O_3_, ozone; ppb, parts per billion.

All models adjusted for age, sex, monthly household income, education, smoking, drinking, physical activity, temperature, humidity.

| Exposure | Model 1^a^ | Model 2^b^ | Model 3^c^ | Model 4^d^ |
| --- | --- | --- | --- | --- |
| PM_2.5_ (µg/m^3^) | 1.19 (1.11-1.26) | 1.19 (1.12-1.27) | 1.29 (1.13-1.46) | 1.19 (1.12-1.27) |
| SO_2_ (ppb) | 1.48 (1.40-1.58) | 1.57 (1.47-1.68) | 2.78 (2.48-3.12) | 1.58 (1.47-1.69) |
| NO_2_ (ppb) | 1.16 (0.73-1.83) | 1.11 (1.03-1.20) | 1.01 (0.86-1.20) | 1.10 (1.02-1.19) |
| CO (ppb) | 1.53 (1.41-1.67) | 1.63 (1.48-1.78) | 7.08 (5.54-9.03) | 1.61 (1.47-1.77) |
| O_3_ (ppb) | 0.71 (0.67-0.76) | 0.48 (0.45-0.52) | 0.26 (0.23-0.29) | 0.48 (0.45-0.52) |

Table S6 Hazard ratios of metabolic syndrome per interquartile range with increment in air pollution exposure during follow-up using different covariates (n=7,428)

Note: IQR for PM_2.5_: 3.48 µg/m^3^, SO_2_: 1.17 ppb, NO_2_: 8.87 ppb, CO: 172.71 ppb, O_3_: 2.65 ppb.

Abbreviations: HR, Hazard ratio; 95% CI, 95% confidence interval; BMI, Body mass index; IQR, Interquartile range; PM_2.5_, particulate matter with aerodynamic diameters ≤ 2.5μm; SO_2_, sulfur dioxide; NO_2_, nitrogen dioxide; CO, carbon monoxide; O_3,_ ozone; ppb, parts per billion.

^a^Model 1 Unadjusted model.

^b^Model 2 adjusted for age, sex, monthly household income, education, smoking, drinking, physical activity, temperature, humidity.

^c^Model 3 adjusted for age, sex, monthly household income, education, smoking, drinking, physical activity, temperature, humidity, region.

^d^Model 4 adjusted for age, sex, monthly household income, education, BMI, smoking, drinking, physical activity, temperature, humidity.


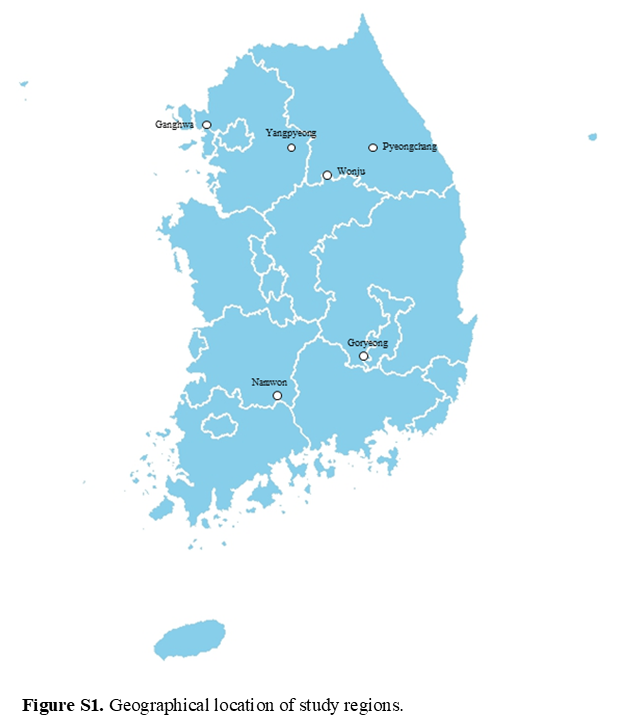


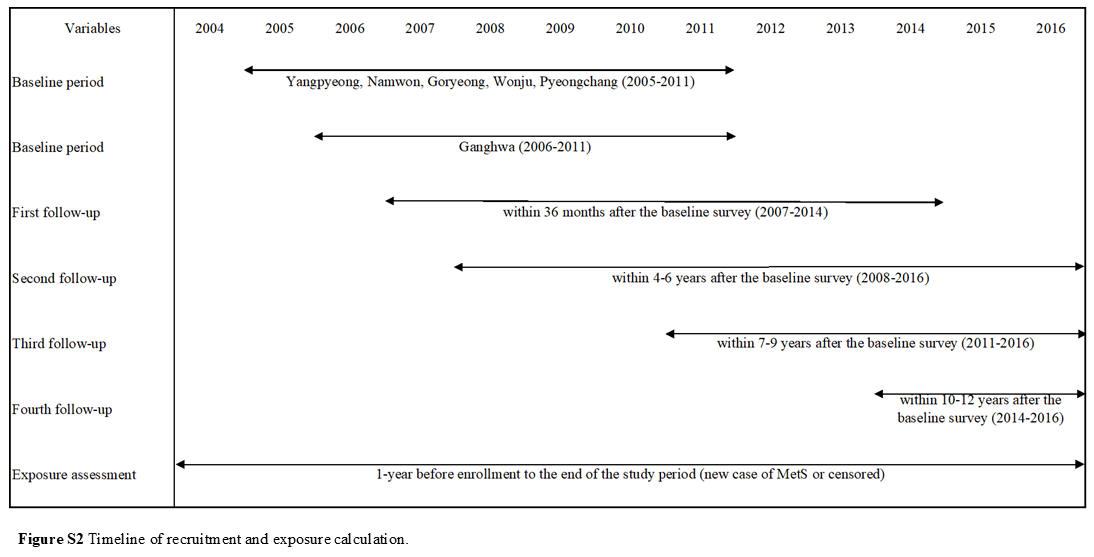


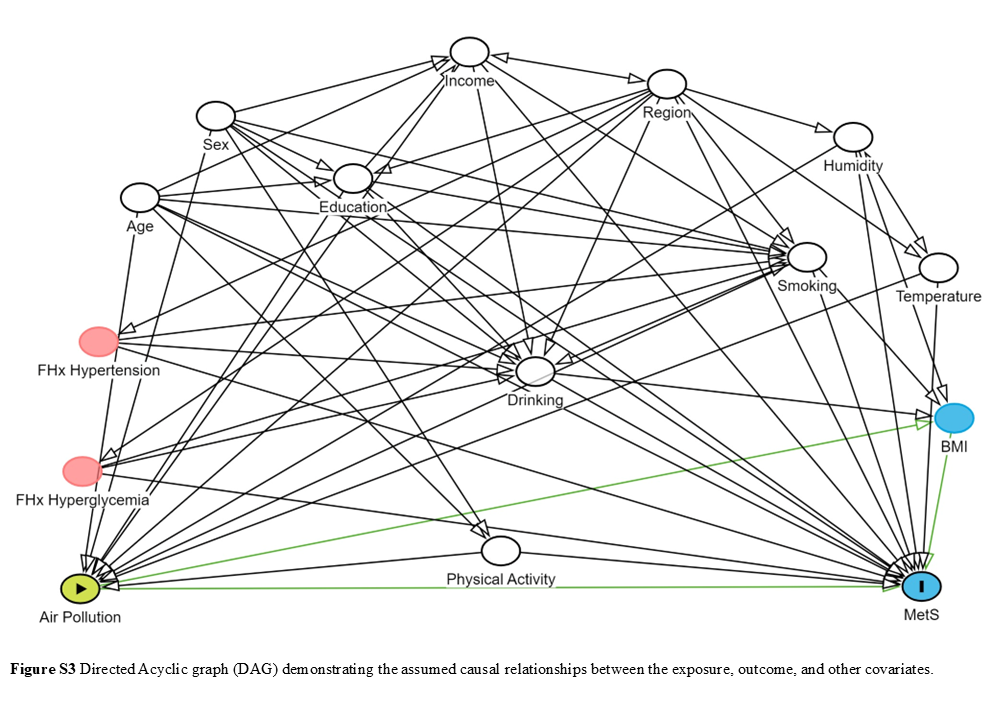


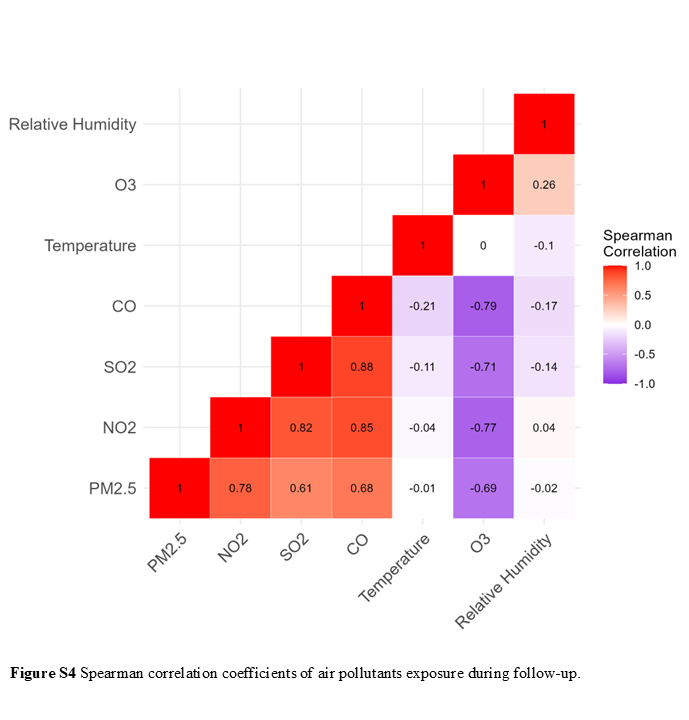

Supplement: Supplementary file 1 — Supplementary Material 1 [file 12940_2025_1158_MOESM2_ESM.docx]
